# Plasma circulating cell‐free *MYCN* gene: A noninvasive and prominent recurrence monitoring indicator of neuroblastoma

**DOI:** 10.1002/cnr2.1688

**Published:** 2022-07-26

**Authors:** Ying Liang, Yan Liu, Pin Zhang, Mengxin Zhang, Bang Du, Weyland Cheng, Zhidan Yu, Lifeng Li, Huanmin Wang, Guangjun Hou, Xianwei Zhang, Wancun Zhang

**Affiliations:** ^1^ Henan Key Laboratory of Children's Genetics and Metabolic Diseases, Zhengzhou Key Laboratory of Precise Diagnosis and Treatment of Children's Malignant Tumors Children's Hospital Affiliated to Zhengzhou University Zhengzhou China

**Keywords:** early, monitor, *MYCN*, neuroblastoma, plasma, relapse

## Abstract

The postoperative recurrence of neuroblastoma (NB) patients is an essential reason for the high mortality of NB due to the lack of early, non‐invasive, and dynamic strategies for monitoring NB recurrence. Therefore, whether the plasma circulating cell‐free *MYCN* gene as an indicator for monitoring of NB recurrence was systematically evaluated. The *MYCN* copy number and *NAGK* (reference gene) copy number (M/N) ratio in plasma and corresponding tumor tissues of NB patients was detected using an economical, sensitive, and specific single‐tube dual RT‐PCR approach developed in this study. The plasma M/N ratio of the *MYCN* gene amplification (MNA) group (*N* = 25, median M/N ratio = 4.90) was significantly higher than that of the non‐MNA group (*N* = 71, median M/N ratio = 1.22), *p* < .001. The M/N ratio in NB plasma (*N* = 60) was positively correlated with the M/N ratio in NB tumor tissue (*N* = 60), with a correlation coefficient of 0.9496. In particular, the results of dynamic monitoring of postoperative plasma M/N ratio of NB patients showed that an abnormal increase in M/N ratio could be detected 1–2 months before recurrence in NB patients. In summary, the single‐tube double RT‐PCR approach can be used to quantitatively detect *MYCN* copy number. The copy number of *MYCN* in the tissue and plasma of NB patients is consistent with each other. More importantly, the circulating cell‐free *MYCN* gene of NB patients can be used as a monitoring indicator for early, non‐invasive, and dynamic monitoring of NB recurrence.

## 1 INTRODUCTION

Neuroblastoma (NB), originating from the postganglionic sympathetic nervous system, is the most common extracranial malignant tumor in children. NB accounts for 8%–10% of pediatric cancer cases and 15% of pediatric tumor deaths.^1^, ^2^, ^3^ NB can be stratified into low‐, intermediate‐, and high‐risk groups based on the demarcation of age at diagnosis, the International Neuroblastoma Staging System (INSS) stage, the tumor tissue *MYCN* status, the International Neuroblastoma Pathology Committee (INPC) classification, and ploidy.^4^ Clinically, most NB patients, especially high‐risk NB, are only diagnosed at an advanced stage due to the poor verbal ability of young NB patients, the hidden location of the tumor and ambiguous early symptoms of NB.^5^ The 5‐year overall survival rate of low‐risk NB and intermediate‐risk NB ranges from 85% to 90%. However, despite intensive multimode therapy used to treat high‐risk NB over the past 30 years, more than 50% of high‐risk NB patients still relapse, resulting in a 5‐year survival rate of less than 10% with a long‐term survival rate of only 2%.^6^, ^7^, ^8^, ^9^, ^10^, ^11^, ^12^ Hence, early diagnosis for the recurrence of high‐risk NB patients is one of the more effective ways to reduce the mortality of high‐risk NB patients. However, there is a lack of clinical methods for the early, noninvasive and dynamic monitoring of recurrence in NB patients. Therefore, it is urgent to establish a non‐invasive and dynamic detection strategy to monitor the recurrence of NB.

The NB tumor tissue *MYCN* gene (*MYCN*) is a widely used clinical biomarker in NB risk grading. *MYCN* gene amplification (MNA) exists in 20%–30% of NB patients, and the overall survival rate in these patients remains below 50%.^13^, ^14^, ^15^ The overexpression of *MYCN* inducing transcriptional activation of *MYCN*, increased *MYCN* protein stability due to dysregulated *MYCN* phosphorylation, and reduced proteasome degradation to *MYCN* gene amplification is closely related to the progression of NB.^16^, ^17^, ^18^ In high‐risk NB patients, *MYCN* amplification, if it occurs, is always present at diagnosis. NB patients with low‐risk disease who lack MNA do not develop high‐risk disease and do not acquire additional copies of *MYCN* gene.^19^ This suggests that MNA is an early and possibly initiating event that drives the progression of high‐risk NB. At present, the status of MNA is determined using either southern blots or fluorescence in situ hybridization (FISH) based on invasive tumor tissue samples. However, these methods are invasive, time‐consuming and expensive and also require a relatively large amount of tumor tissue. In particular, NB is highly heterogeneous, which may result in deviation of test results and is not representative of the overall tumor phenotype.^20^, ^21^, ^22^, ^23^, ^24^, ^25^, ^26^ Therefore, it is necessary to establish a non‐invasive, rapid, sensitive and specific diagnostic method of *MYCN* status for the early diagnosis and recurrence detection of NB. Iehara, Ma, Combaret and Gotoh, have shown that plasma circulating cell‐free *MYCN* in MNA NB patients is higher than in non‐MNA NB.^27^, ^28^, ^29^, ^30^ However, these studies did not conduct the following experiments: (1) the *MYCN* copy number in NB tumor tissue was not detected quantitatively; (2) Whether the plasma *MYCN* copy number can dynamically monitor the NB recurrence was not systematically studied. Therefore, the *MYCN* copy number in NB plasma and tumor tissue was systematically examined in this study.

In order to accurately quantify the *MYCN* copy number in plasma and tumor tissue of NB patients, the N‐acetylglucosamine kinase gene (*NAGK*) was selected as the internal reference gene. NAGK is located on the same chromosome as *MYCN* but is sufficiently distanced from the *MYCN* amplicon region. The ratio of *MYCN* copy number to *NAGK* copy number (*MYCN*/*NAGK*, M/N) was used to assess the amplification of *MYCN* copy number. To further accurately quantify M/N ratio, a highly sensitive and specific single‐tube multiplex RT‐PCR approach was developed using a *MYCN* molecular beacon (MB) and *NAGK* MB to detect *MYCN* PCR products and *NAGK* PCR amplification products, respectively. Subsequently, the plasma and tumor tissue of NB was systematically studied using the developed single‐tube multiplex RT‐PCR approach. In particular, we dynamically monitored postoperative plasma *MYCN* copy number in NB patients to further evaluate the feasibility of plasma circulating cell‐free *MYCN* as a noninvasive indicator of NB recurrence using the developed single‐tube multiplex RT‐PCR approach. The innovations of this study are summarized as follows. (1) A highly sensitive and specific single‐tube multiplex RT‐PCR approach was developed to detect the plasma and tumor tissue M/N ratio. (2) The consistency of M/N ratio in plasma and tissue of NB patients was systematically evaluated. (3) The feasibility of using M/N ratio in plasma of MNA NB patients for non‐invasive and dynamic monitoring of recurrence in NB patients was studied. This study is expected to provide theoretical support for early and non‐invasive recurrence monitoring of MNA NB.

## 2 MATERIALS AND METHODS

### 2.1 Patients

The plasma and tumor tissue samples of NB patients and corresponding clinical data were collected from Children's Hospital Affiliated to Zhengzhou University. Inclusion criteria are as follows: (1) histologically confirmed NB diagnosis; (2) assessment of MNA status by FISH method; (3) availability of tumor and plasma samples; (4) written informed consent from patients or their parents at the time of sample collection. The research proposal was approved by the ethics review committee of Children's Hospital Affiliated to Zhengzhou University.

### 2.2 Reagents, materials and instruments

All DNA used in this study was purchased from Sangon Biotech (Shanghai, China) and all sequences are listed in Table S1. Diethyl pyrocarbonate (DEPC) treated water, and dNTP were purchased from TaKaRa Biotechnology Co. Ltd. (Dalian, China; DEPC, diethylpyrocarbonate). 2 × Ace Taq Master Mix was purchased from Vazyme Biotech Co., Ltd (Nanjing, China). The real‐time fluorescence measurements were performed with a Biorad CFX Opus 96 Real Time PCR System (Richmond, USA). An OD‐1000^+^ UV–Vis spectrophotometer (One Drop® Technologies, China) was used for the absolute quantification of DNA. All chemicals and solvents were of analytical grade purity and were purchased from Aladdin (Shanghai, China).

### 2.3 Sample preparation

Tumor specimens were surgically removed and immediately stored in liquid nitrogen. To avoid contamination of plasma DNA by white blood cell (WBC) DNA, whole blood of patients with NB was centrifuged at 3000 r/min for 10 min within 2 h, plasma was separated and stored at −80°C until DNA extraction.

### 2.4 Cell culture and DNA Isolation

SK‐N‐BE2, SK‐N‐AS, SH‐SY5Y and HUVEC cell lines were cultured in DMEM supplemented with 10% FBS, penicillin (100 μg/ml) and streptomycin (100 μg/ml) in 5% CO_2_ at 37°C. Total extraction kit DP304 purchased from Tiangen Biotech (Beijing China) was used to extract the total DNA in cell lines NB tissue samples and plasma specimens.

### 2.5 Single tube duplex RT‐PCR

The single tube duplex RT‐PCR was conducted by the following method. 5 μl of 2 × Ace Taq Master Mix, 0.4 μl of *MYCN* and *NAGK* forward primer (10 μM), 0.4 μl of *MYCN* and *NAGK* reverse primer (10 μM), 0.5 μl of *MYCN* and *NAGK* MB (10 μM), 1 μl of synthetic target DNA or total DNA extracted from real samples and 2.7 μl DEPC water were mixed to a final volume of 10 μl. The RT‐PCR was conducted under the following conditions: 95°C for 5 min, 40 cycles of 95°C for 10 s, 28°C for 30 s and 60°C for 30 s. The fluorescence signal was detected at 28°C.

### 2.6 Statistical methods

Statistical analysis was performed using SPSS 21.0 software.

## 3 RESULTS AND DISCUSSION

### 3.1 Feasibility and sensitivity of the single‐tube duplex RT‐PCR

**FIGURE 1:**
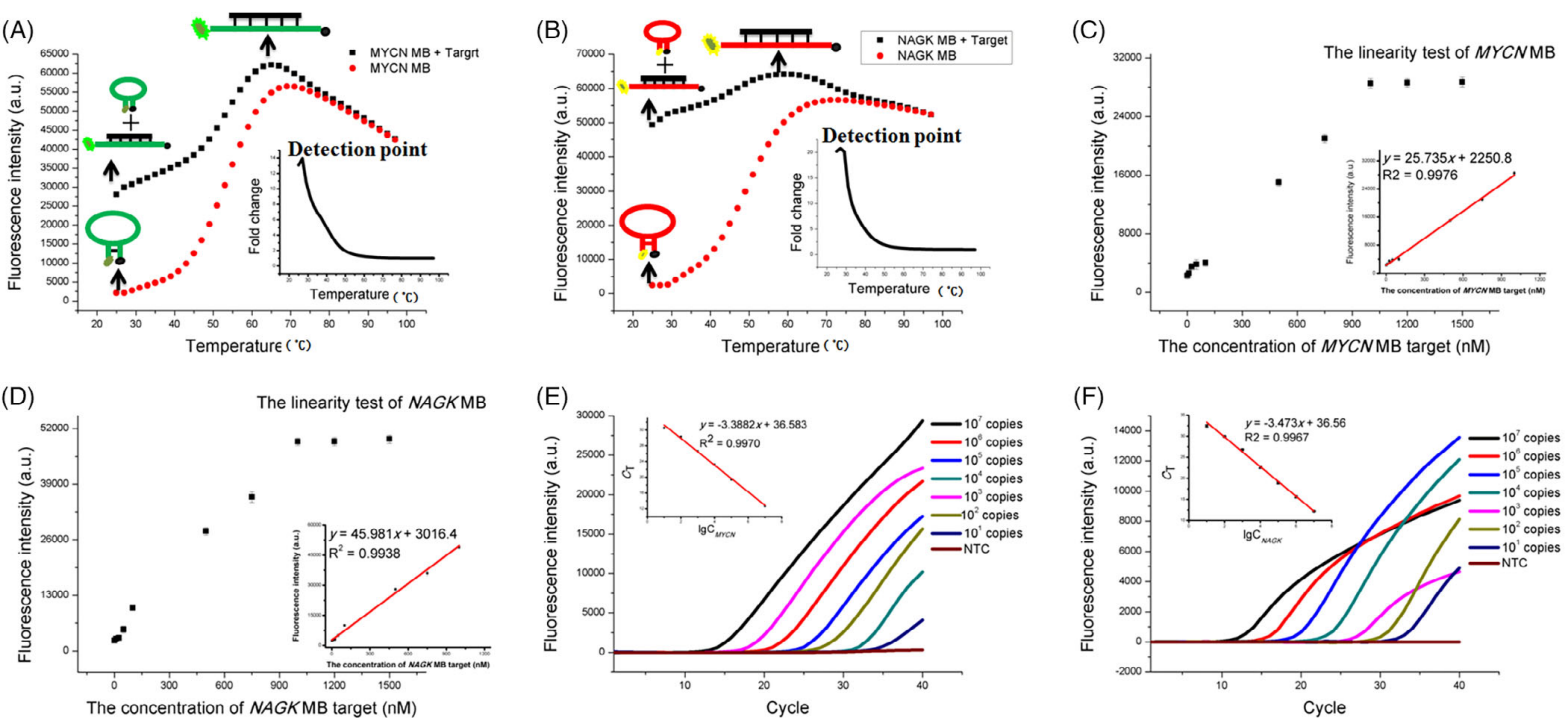
(A) The fluorescence signal changes of *MYCN* MB in the presence and absence of the target sequence by increasing the temperature from 25 to 95°C. Inset: the fluorescence signal fold change of *MYCN* MB varies from 25 to 95°C in the presence and absence target sequence. (B) The fluorescence signal changes of *NAGK* MB in the presence and absence of the target sequence by increasing the temperature from 25 to 95°C. Inset: the fluorescence signal fold change of *NAGK* MB varies from 25 to 95°C in the presence and absence of target sequence. (C) The relationship between the fluorescence value of *MYCN* MB and the target sequence. Inset: calibration curve of *MYCN* MB fluorescence value with the target sequence. (D) The relationship between the fluorescence value of *NAGK* MB and the target sequence. Inset: calibration curve of *NAGK* MB fluorescence value with the target sequence. (E) Real‐time fluorescence curves of the response of the developed single tube duplex RT‐PCR to different copy numbers of *MYCN*. Inset: the *C*_T_ values of the developed single tube duplex RT‐PCR show a logarithmic linear correlation with the *MYCN* copy number. (F) Real‐time fluorescence curves of the developed single tube duplex RT‐PCR for *NAGK* response with different copy numbers. Inset: The *C*_T_ values of the developed single tube duplex RT‐PCR show a logarithmic linear correlation with the *NAGK* copy number.

The feasibility of single‐tube duplex RT‐PCR, using two‐color MB (*MYCN* MB and *NAGK* MB) to detect *MYCN* and *NAGK* in one PCR tube, was systematically studied. First, to evaluate the feasibility of the *MYCN* MB and *NAGK* MB in quantifying their target sequence, the features of MBs were studied. The secondary structure of *MYCN* MB and *NAGK* MB were predicted by Quikfold Fast Folding (http://www.unafold.org/Dinamelt/applications/quickfold.php), which both possessed a stem‐loop structure (Figure S1A,B). The *MYCN* MB and *NAGK* MB were modified at their 5′ end with a fluorophore (FAM and VIC, respectively) and at their 3′ end with a quencher (BHQ‐1). The fluorescence values of *MYCN* MB and *NAGK* MB were detected in the presence and absence of the target sequence, and the optimal reaction temperature of *MYCN* MB and *NAGK* MB were both 28°C (Figure 1A,B). The fold change value is the value of the fluorescence of MB in the presence and absence of target sequence. The optimum reaction temperature for *MYCN* MB and *NAGK* MB were both 28°C (Figure 1A,B). We then investigated the sensitivity of MB to detect target sequences. When the concentration of *MYCN* MB was 1000 nM, the MB fluorescence signal was linearly related to the target concentration (with a range from 10 to 1000 nM). The correlation equation was *y* = 25.735 *x* + 2250.8 (*R* ^2^ = 0.9976), where, *y* is the fluorescence. The fold change value is the value of the fluorescence of MB in the presence and absence of target sequence of *MYCN* MB and *x* is the concentration of target sequence (Figure 1C). Meanwhile, *NAGK* MB fluorescence signal is linearly related to target concentration. The correlation equation was *y* = 45.981 *x* + 3016.4 (*R* ^2^ = 0.9938) where *y* is the fluorescence value of *NAGK* MB, *x* is the concentration of target sequence (Figure 1D). Those results demonstrated that *MYCN* MB and *NAGK* MB have high sensitivity to detect the target sequence. In conclusion, the above experimental results demonstrated the feasibility of the designed MB to detect target sequences.

Sensitivity and specificity are important factors in evaluating the developed approaches due to the lower content of *MYCN* in plasma and tissue. To test whether a single‐tube duplex RT‐PCR method could effectively detect *MYCN* and *NAGK* quantitatively, experiments were carried out with the results depicted in Figure 1E,F. Figure 1E shows the real‐time fluorescence curves of different *MYCN* copy numbers where the *C*_T_ values increase with a decrease in *MYCN* copy numbers. On the logarithmic scale, *C*_T_ values and *MYCN* copy numbers yielded a good linear relationship on seven orders of magnitude from 10 to 10^7^ copies. The correlation equation is *y* = −3.3882 lg *x* + 36.583 (*y* and *x* are the *C*_T_ value and *NAGK* copy number, respectively) and the correlation coefficient is *R* ^2^ = 0.9970. Figure 1F shows the real‐time fluorescence curve of different *NAGK* copy numbers where the *C*_T_ value increases with a decrease in *NAGK* copy number. On the logarithmic scale, *C*_T_ values and *NAGK* copy numbers yielded a good linear relationship on seven orders of magnitude from 10 to 10^7^ copies. The correlation equation is *y* = −3.473 lg *x* + 36.56 (*y* and *x* are the *C*_T_ value and *NAGK* copy number, respectively) and the correlation coefficient is *R* ^2^ = 0.9967. Therefore, the developed single‐tube duplex RT‐PCR can quantitatively detect *MYCN* and *NAGK* in a single tube with high sensitivity.

### 3.2 Specificity and reproducibility of the single‐tube duplex RT‐PCR

**FIGURE 2:**
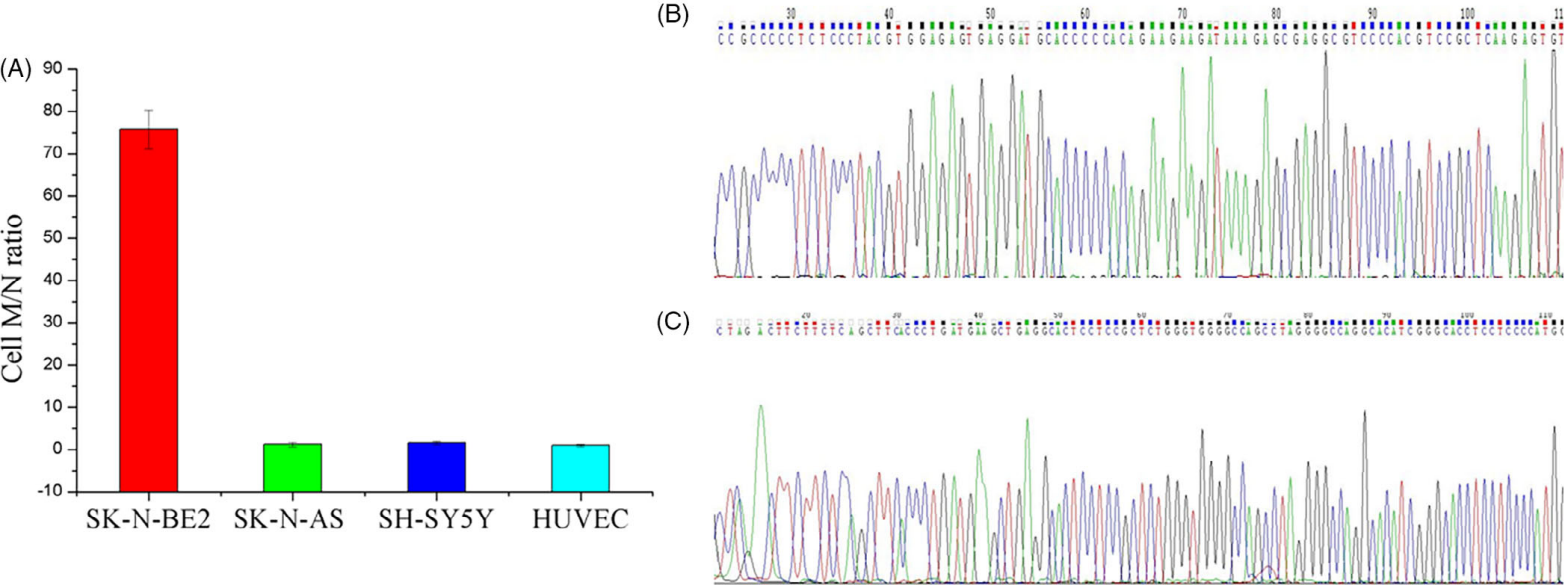
(A) The M/N ratio in SK‐N‐AS, SK‐N‐BE2, SH‐SY5Y and HUVEC. The representative sequencing results of (B) *MYCN* PCR product and (C) *NAGK* PCR product

Due to the complexity of clinical samples, specificity is an important indicator for evaluating and establishing detection approaches. In order to evaluate the specificity of the established single‐tube duplex RT‐PCR, this study used single‐tube duplex RT‐PCR to detect the M/N ratio of one MNA NB cell line (SK‐N‐BE2), two non‐MNA NB cell line (SK‐N‐AS, SH‐SY5Y) and one human umbilical vein endothelial cell (HUVEC), indicating that the M/N ratio of SK‐N‐BE2 is higher than SK‐N‐AS, SH‐SY5Y and HUVEC. The M/N ratio of SK‐N‐AS, SH‐SY5Y and HUVEC is less than 2 (Figure 2A). Furthermore, the amplification products of *MYCN* and *NAGK* in NB tissues were sequenced based on the single‐tube double RT‐PCR method and the sequencing results were more than 99% similar to the target sequences (Figure 2B,C). Therefore, the single‐tube double RT‐PCR method established in this study has high specificity in real sample analysis. In addition, the reproducibility of the developed single‐tube duplex RT‐PCR recipe was investigated by five consecutive assays where the RSDs for *MYCN* and *NAGK* were determined to be 1.3% and 0.7%, respectively (Figure S3). The results show that the developed single‐tube duplex RT‐PCR recipe has acceptable reproducibility. In summary, the developed approach has high specificity in real sample analysis and acceptable reproducibility.

### 3.3 The M/N ratio in NB tumor tissue and plasma

**FIGURE 3:**
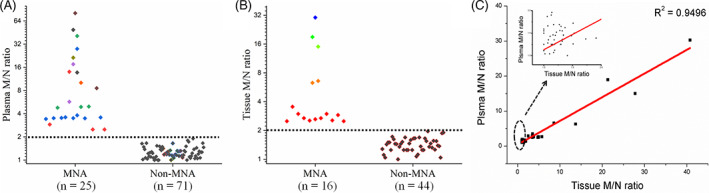
(A) The plasma M/N ratio of MNA NB and non‐MNA NB. (B) The tumor tissue M/N ratio in MNA NB and non‐MNA NB. The middle line represents the cut‐off level (2.0). (C) Consistency of NB tissue M/N ratio and plasma M/N ratio

The total DNA was extracted from 96 cases of NB plasma samples and 60 of cases NB clinical samples of NB tumor tissues, and the *MYCN* copy number in plasma and tissues. The OD_260_/OD_280_ of total DNA extracted from plasma and tissue specimens were detected with a range from 1.80 to 1.90, which indicated that the extracted DNA was of good quality (Table S2). First, the *MYCN* amplification status of these 96 cases of NB tumor tissues was detected by FISH, and the 96 cases of NB were subsequently divided into an MNA group (*n* = 25) and non‐MNA group (*n* = 71) according to MNA status. There were no statistically significant differences between the MNA group and the non‐MNA group in terms of gender, age, and stage of diagnosis (Table 1). Following, the M/N ratio of corresponding plasma (*n* = 96) was detected by single‐tube duplex RT‐PCR approach. The experimental results showed that the M/N values in the plasma of the MNA group (*n* = 25) were all greater than 2 whereas the M/N values in the plasma of the non‐MNA group (*n* = 71) were all less than 2 (Figure 3A and Table 1). The plasma M/N ratio in the MNA group (*n* = 25, median M/N ratio = 4.90) was significantly higher than that in the non‐MNA group (*n* = 71, median M/N ratio = 1.22), *p* < .001 (Figure S2A). Meanwhile, the M/N ratio in 60 cases of NB tumor tissues, including 16 MNA cases and 44 non‐MAN cases, was detected by single‐tube duplex RT‐PCR approach. The experimental results also showed that the M/N ratios in MNA group (*n* = 16) were greater than 2 and the M/N ratios in non‐MNA group (*n* = 16) were less than 2 (Figure 3B and Table 1). The M/N ratios in the tissue of the MNA group (*n* = 16, median M/N ratio = 2.94) were significantly higher than that of the non‐MNA group (*n* = 71, median M/N ratio = 1.44), *p* < .05 (Figure S2B). When the M/N ratio cut‐off value was set to 2, the detection results of the 96 cases of plasma and 60 tumor tissues based on the detection method established in this study were completely consistent with the detection results of FISH. Lastly, the M/N ratio in 60 cases of NB with both plasma and tissue samples was detected by the developed single‐tube duplex RT‐PCR approach. Experimental results showed that the correlation coefficient between plasma M/N ratio and tumor tissue M/N ratio was 0.9496 (Figure 3C). Therefore, the M/N ratios in NB plasma and tumor tissues are consistent with each other.

**TABLE 1:** Characteristics of patients according to plasma and tissue *MYCN* status

	Plasma			Tissue		
	MNA	non‐MNA	*p*	MNA	non‐MNA	*p*
Sex, no. (%)			.304			.481
Male	16 (0.64)	37 (0.52)		10 (0.63)	23 (0.52)	
Female	9 (0.36)	34 (0.48)		6 (0.37)	21 (0.48)	
Median age at diagnosis (rang)	17.00 (1.87–120.00)	36.00 (10.00–168.00)	0.061	20.00 (1.87–120.00)	36.00 (2.20–168.00)	0.265
Stage, no. (%)			.384			.129
Low risk	4 (0.16)	16 (0.23)		0 (0.00)	9 (0.20)	
Medium risk	4 (0.16)	18 (0.25)		4 (0.25)	11 (0.25)	
High risk	17 (0.68)	37 (0.52)		12 (0.75)	24 (0.55)	
Transfer area, no. (%)			.195			.167
Bone or bone marrow	12 (0.48)	33 (0.46)		11 (0.69)	21 (0.48)	
Lymph nodes	6 (0.24)	14 (0.56)		4 (0.25)	10 (0.22)	
Other parts	3 (0.12)	2 (0.03)		1 (0.06)	2 (0.05)	
No transfer	4 (0.16)	22 (0.31)		0	11 (0.25)	
*MYCN* PCR results median (range)	4.90 (2.50–80.79)	1.22 (1.00–1.96)	.000	2.94 (2.50–30.29)	1.44 (1.00–1.96)	.017

### 3.4 Dynamic detection of M/N ratio in NB plasma

**FIGURE 4:**
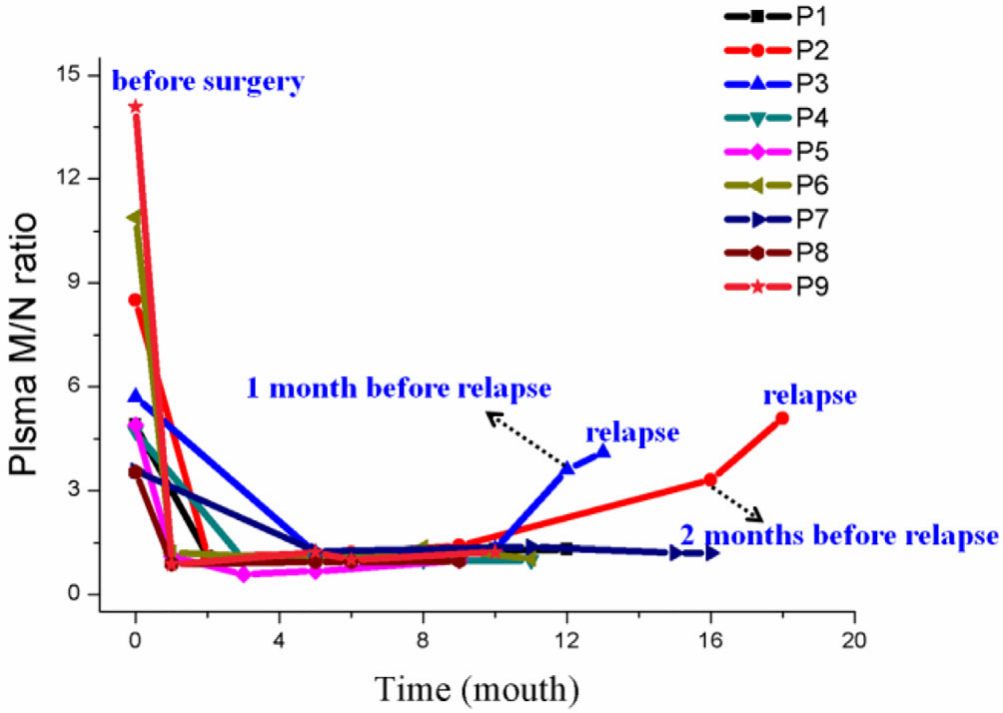
Monitoring the plasma M/N ratio of 9 MNA NB patients

The postoperative recurrence of NB patients is one of the key reasons for the high mortality of NB. Therefore, there is an urgent need to develop early and dynamic monitoring methods for the recurrence of NB. The clinical significance of *MYCN* in monitoring recurrence in NB patients was evaluated by dynamically monitoring the plasma M/N ratios of 9 patients with MNA cases after surgery. As shown in Figure 4, the plasma M/N ratio of 9 MNA NB patients dropped below 2.0 after surgical treatment, which is consistent with the M/N ratio of non‐MNA amplified tumor patients. In addition, we found that P2 and P9 had M/N ratios greater than 2.0 1–2 months before relapse. Clinical data of two relapsed patients are shown in Table S3. At the same time, we compared the strategies for detecting MNA status (Table S4). Therefore, circulating free *MYCN* in NB plasma is expected to be an early, non‐invasive, and dynamic monitoring indicator for relapse in NB patients, which needs to be further demonstrated by larger cohort studies.

## 4 CONCLUSIONS

A rapid, sensitive, and specific single‐tube dual RT‐PCR approach was developed for accurate quantification of NB plasma and tumor tissue M/N ratio. The M/N ratio in NB plasma and tumor tissue detected by single‐tube dual RT‐PCR approach were consistent with each other. Meanwhile, the M/N ratio in NB tumor tissue detected by single‐tube dual RT‐PCR approach was consistent with FISH results. By dynamically monitoring 9 MNA NB after surgery, we found the M/N ratio greater than cut‐off value before 1–2 months, illustrating that the plasma *MYCN* amplification status can be used as a non‐invasive indicator of NB recurrence, although this conclusion requires more NB recurrences case proof. In summary, plasma circulating free *MYCN* gene is expected to serve as a non‐invasive and prominent indicator for monitoring NB recurrence.

## AUTHOR CONTRIBUTIONS

*Data curation, methodology, writing—original draft*, Y. Liang; *Data curation*, Y. Liu; *Formal analysis*, P.Z.; *Investigation*, M.Z.; *Investigation*, B.D.; *Software*, W.C.; *Supervision*, Z.Y.; *Visualization*, L.L.; *Data curation*, H.W.; *Writing—review and editing*, G.H.; *Writing—review and editing*, X.Z.; *Funding acquisition, project administration, supervision, writing—review and editing*, W.Z.

## CONFLICT OF INTEREST

The authors declare no conflict of interest.

## ETHICS STATEMENT

The research proposal was approved by the ethics review committee of Children's Hospital Affiliated to Zhengzhou University.

## Supporting information


**Appendix S1** Supporting Information

## Data Availability

The data that support the findings of this study are available from the corresponding author upon reasonable request.
