# Supplementary material for: Plasma circulating cell‐free MYCN gene: A noninvasive and prominent recurrence monitoring indicator of neuroblastoma
Source: Cancer Rep (Hoboken). 2022 Jul 26;6(2):e1688. doi: 10.1002/cnr2.1688 (PMC9939986; doi:10.1002/cnr2.1688)
Supplement: Supplementary file 1 — Appendix S1 Supporting Information [file CNR2-6-e1688-s001.docx]

**Plasma circulating cell-free *MYCN* gene: a noninvasive and prominent recurrence monitoring indicator of neuroblastoma**

Ying Liang, Yan Liu, Pin Zhang, Mengxin Zhang, Bang Du, Weyland Cheng, Zhidan Yu, Lifeng Li, Huanmin Wang, Guangjun Hou*, Xianwei Zhang*, Wancun Zhang*

Henan Key Laboratory of Children’s Genetics and Metabolic Diseases, Zhengzhou Key Laboratory of Precise Diagnosis and Treatment of Children’s Malignant Tumors, Children’s Hospital Affiliated to Zhengzhou University, Zhengzhou, 450018, China

^*^ Correspondence to:

Wancun Zhang, PhD, Xianwei Zhang, PhD and Guangjun Hou Professor, Henan Key Laboratory of Children’s Genetics and Metabolic Diseases, Zhengzhou Key Laboratory of Precise Diagnosis and Treatment of Children’s Malignant Tumors, Children’s Hospital Affiliated to Zhengzhou University, longhu road 33,

Tel.: 86-373-63866536,

Email: zhangwancun@126.com (Wancun Zhang); zhangxw956658@126.com (Xianwei Zhang); [houguangjun2022@126.com](mailto:houguangjun2022@126.com) (Guangjun Hou)

**Table S1** The sequences for *MYCN* approach and *NAGK* approach.

| Gene | Name | Sequence（5’ → 3’） |
| --- | --- | --- |
| *MYCN* | Forward primer | AGCGA GCTGA TCCTC AAACG |
|  | Reverse primer | GGGAT GACAC TCTTG AGCGG |
|  | Molecular beacon | FAM - CGCGA TGATG CACCC CCACA GAAGA AGATA AAGAG ATCGC G - BHQ1 |
| *NAGK* | Forward primer | TTTCT TCTGG CGCTG ACCC |
|  | Reverse primer | CGCTA TAGTC CATGG GGAG |
|  | Molecular beacon | VIC - CGCGA TTCTT CTCCA GCTTC ACCCT GATGT CGCG - BHQ1 |


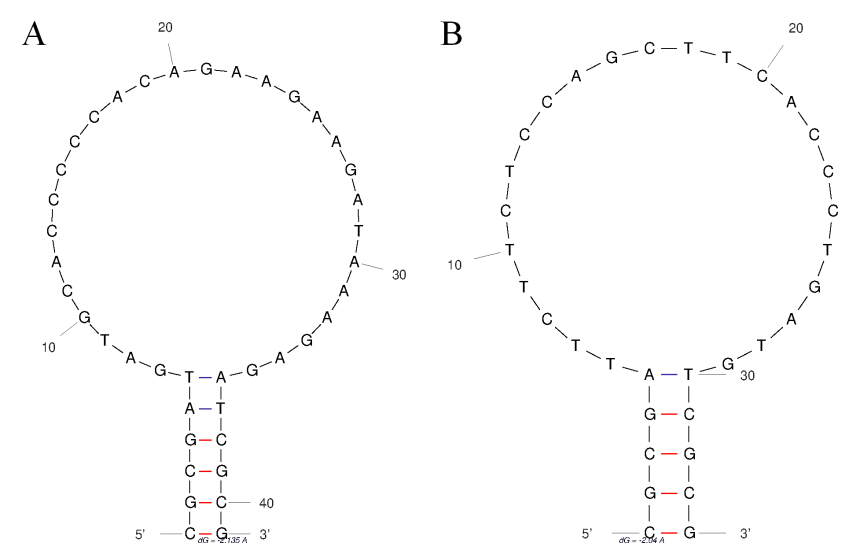


**Figure S1** The secondary structure of (A) *MYCN* MB and (B) *NAGK* MB.


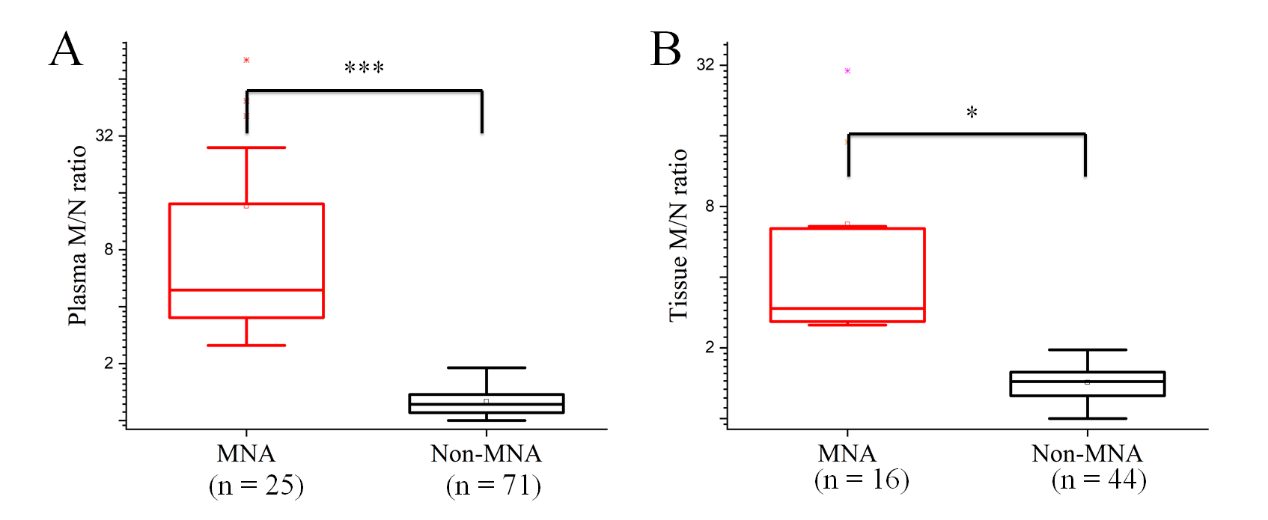


**Figure S2** (A) Concentrated distribution of plasma M/N ratios. (B) Centralized distribution of tissue M/N ratios.


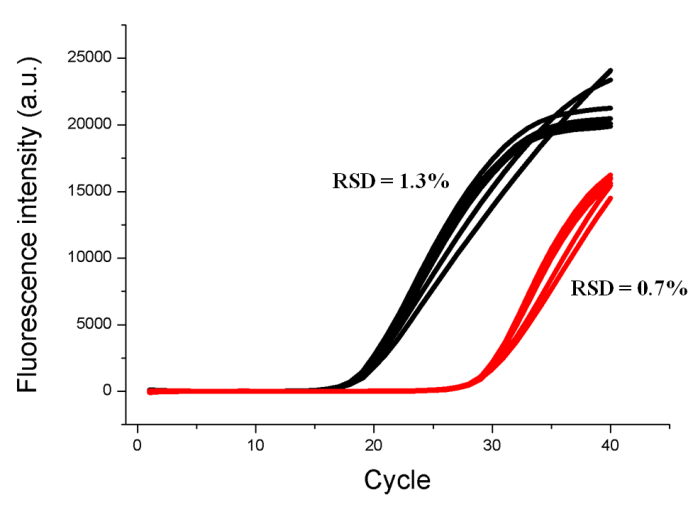


**Figure S3** The repeatability of developed *MYCN* approach

**Table S2** The DNA extraction results and M/N ratios of clinical samples

| **Plasma** | | | | **Tissue** | | | |
| --- | --- | --- | --- | --- | --- | --- | --- |
| NO. | Content (ng/μL) | OD_260_/OD_280_ | M/N ratio | NO. | Content (ng/μL) | OD_260_/OD_280_ | M/N ratio |
| S35 | 2.80 | 1.85 | 1.18 | S35 | 38.30 | 1.83 | 1.35 |
| S37 | 4.30 | 1.80 | 1.06 | S37 | 68.30 | 1.85 | 1.87 |
| S40 | 3.50 | 1.83 | 8.58 | S40 | 207.6 | 1.89 | 6.58 |
| S49 | 5.15 | 1.88 | 1.02 | S49 | 106.7 | 1.92 | 1.04 |
| S112 | 4.70 | 1.84 | 1.37 | S112 | 46.40 | 1.83 | 1.62 |
| S114 | 5.40 | 1.80 | 13.71 | S114 | 53.40 | 1.88 | 6.30 |
| S139 | 2.40 | 1.82 | 1.18 | S139 | 39.20 | 1.82 | 1.25 |
| S142 | 4.90 | 1.88 | 1.10 | S142 | 246.50 | 1.88 | 1.04 |
| S143 | 7.90 | 1.87 | 1.00 | S143 | 17.60 | 1.81 | 1.59 |
| S145 | 9.10 | 1.88 | 1.19 | S145 | 19.10 | 1.84 | 1.12 |
| S146 | 4.50 | 1.85 | 1.14 | S146 | 184.10 | 1.87 | 1.96 |
| S148 | 3.20 | 1.81 | 1.48 | S148 | 42.70 | 1.83 | 1.44 |
| S159 | 2.30 | 1.80 | 1.28 | S159 | 19.50 | 1.82 | 1.56 |
| S169 | 4.20 | 1.85 | 1.00 | S169 | 70.20 | 1.82 | 1.50 |
| S176 | 5.70 | 1.80 | 40.76 | S176 | 23.00 | 1.83 | 30.29 |
| S200 | 3.30 | 1.84 | 1.17 | S200 | 10.80 | 1.87 | 1.69 |
| S216 | 4.80 | 1.88 | 1.13 | S216 | 37.90 | 1.83 | 1.25 |
| S229 | 3.80 | 1.82 | 3.50 | S229 | 50.30 | 1.92 | 2.54 |
| S230 | 6.50 | 1.89 | 1.19 | S230 | 57.80 | 1.82 | 1.25 |
| S232 | 4.10 | 1.85 | 1.07 | S232 | 410.00 | 1.87 | 1.66 |
| S235 | 3.50 | 1.83 | 1.07 | S235 | 158.10 | 1.86 | 1.28 |
| S298 | 6.40 | 1.86 | 1.51 | S298 | 54.90 | 1.86 | 1.72 |
| S318 | 6.50 | 1.85 | 1.00 | S318 | 130.30 | 1.83 | 1.11 |
| S331 | 7.40 | 1.87 | 1.30 | S331 | 140.20 | 1.86 | 1.25 |
| S337 | 3.90 | 1.82 | 27.77 | S337 | 30.00 | 1.82 | 15.04 |
| S341 | 8.60 | 1.85 | 1.11 | S341 | 113.00 | 1.83 | 1.43 |
| S342 | 3.50 | 1.80 | 1.00 | S342 | 53.30 | 1.89 | 1.55 |
| S346 | 6.00 | 1.82 | 1.32 | S346 | 18.00 | 1.80 | 1.32 |
| S348 | 3.70 | 1.87 | 3.48 | S348 | 34.20 | 1.82 | 2.98 |
| S349 | 10.20 | 1.86 | 21.33 | S349 | 182.40 | 1.82 | 18.97 |
| S350 | 6.40 | 1.82 | 1.27 | S350 | 83.70 | 1.87 | 1.69 |
| S355 | 2.60 | 1.87 | 1.34 | S355 | 33.50 | 1.85 | 1.46 |
| S366 | 5.30 | 1.80 | 1.15 | S366 | 99.90 | 1.86 | 1.25 |
| S374 | 3.20 | 1.84 | 1.16 | S374 | 463.40 | 1.86 | 1.46 |
| S380 | 4.10 | 1.82 | 3.53 | S380 | 32.60 | 1.81 | 3.54 |
| S386 | 0.50 | 1.85 | 1.91 | S386 | 49.80 | 1.86 | 1.39 |
| S398 | 3.60 | 1.86 | 1.13 | S398 | 27.70 | 1.83 | 1.90 |
| S405 | 3.70 | 1.85 | 1.20 | S405 | 34.60 | 1.83 | 1.45 |
| S408 | 5.10 | 1.87 | 3.50 | S408 | 20.10 | 1.88 | 2.50 |
| S409 | 3.80 | 1.84 | 4.78 | S409 | 19.70 | 1.81 | 2.50 |
| S411 | 4.20 | 1.83 | 1.08 | S411 | 269.40 | 1.81 | 1.35 |
| S412 | 7.20 | 1.88 | 1.32 | S412 | 32.30 | 1.89 | 1.55 |
| S415 | 6.70 | 1.81 | 3.81 | S415 | 13.20 | 1.82 | 2.62 |
| S423 | 5.70 | 1.89 | 1.32 | S423 | 24.30 | 1.83 | 1.36 |
| S430 | 3.80 | 1.85 | 1.29 | S430 | 113.00 | 1.85 | 1.25 |
| S431 | 3.50 | 1.89 | 1.41 | S431 | 19.20 | 1.80 | 1.00 |
| S437 | 4.50 | 1.82 | 5.72 | S437 | 116.80 | 1.88 | 2.69 |
| S448 | 3.90 | 1.81 | 1.17 | S448 | 22.90 | 1.86 | 1.07 |
| S453 | 5.00 | 1.82 | 1.30 | S453 | 261.10 | 1.87 | 1.65 |
| S457 | 4.00 | 1.87 | 1.34 | S457 | 26.90 | 1.82 | 1.45 |
| S459 | 2.50 | 1.86 | 2.5 | S459 | 19.40 | 1.87 | 2.90 |
| S460 | 4.40 | 1.81 | 1.73 | S460 | 13.30 | 1.89 | 1.37 |
| S463 | 3.40 | 1.82 | 3.6 | S463 | 15.10 | 1.82 | 2.98 |
| S470 | 4.70 | 1.90 | 1.53 | S470 | 31.00 | 1.86 | 1.50 |
| S480 | 3.70 | 1.82 | 4.9 | S480 | 13.40 | 1.85 | 2.69 |
| S483 | 5.20 | 1.81 | 4.96 | S483 | 145.00 | 1.89 | 2.55 |
| S485 | 4.20 | 1.81 | 1.00 | S485 | 145.00 | 1.89 | 1.25 |
| S519 | 2.80 | 1.82 | 1.51 | S519 | 34.60 | 1.82 | 1.26 |
| S521 | 4.40 | 1.89 | 1.65 | S521 | 209.10 | 1.81 | 1.65 |
| S525 | 2.80 | 1.87 | 1.22 | S525 | 25.50 | 1.86 | 1.52 |
| S000 | 2.40 | 1.86 | 1.44 | / | / | / | / |
| S090 | 5.20 | 1.83 | 1.38 | / | / | / | / |
| S098 | 4.50 | 1.80 | 1.12 | / | / | / | / |
| S099 | 3.80 | 1.83 | 1.66 | / | / | / | / |
| S113 | 5.80 | 1.89 | 1.10 | / | / | / | / |
| S124 | 3.60 | 1.81 | 1.48 | / | / | / | / |
| S177 | 6.20 | 1.87 | 1.22 | / | / | / | / |
| S191 | 3.40 | 1.82 | 1.30 | / | / | / | / |
| S208 | 4.40 | 1.82 | 1.27 | / | / | / | / |
| S263 | 6.40 | 1.89 | 49.09 | / | / | / | / |
| S290 | 5.70 | 1.87 | 1.66 | / | / | / | / |
| S315 | 3.90 | 1.86 | 1.27 | / | / | / | / |
| S323 | 2.40 | 1.80 | 1.20 | / | / | / | / |
| S333 | 3.20 | 1.81 | 1.05 | / | / | / | / |
| S357 | 3.40 | 1.82 | 1.23 | / | / | / | / |
| S371 | 5.60 | 1.85 | 1.23 | / | / | / | / |
| S375 | 4.60 | 1.81 | 1.13 | / | / | / | / |
| S388 | 3.20 | 1.80 | 1.38 | / | / | / | / |
| S403 | 4.80 | 1.85 | 17.56 | / | / | / | / |
| S410 | 2.50 | 1.88 | 14.01 | / | / | / | / |
| S418 | 6.20 | 1.83 | 1.28 | / | / | / | / |
| S449 | 6.20 | 1.80 | 1.07 | / | / | / | / |
| S461 | 8.60 | 1.86 | 1.47 | / | / | / | / |
| S464 | 4.80 | 1.80 | 80.79 | / | / | / | / |
| S473 | 2.80 | 1.80 | 1.25 | / | / | / | / |
| S478 | 3.80 | 1.86 | 1.64 | / | / | / | / |
| S479 | 3.70 | 1.83 | 10.09 | / | / | / | / |
| S484 | 5.70 | 1.82 | 1.07 | / | / | / | / |
| S490 | 7.60 | 1.98 | 3.42 | / | / | / | / |
| S507 | 6.40 | 1.81 | 2.90 | / | / | / | / |
| S509 | 6.10 | 1.86 | 3.58 | / | / | / | / |
| S510 | 5.90 | 1.80 | 1.02 | / | / | / | / |
| S516 | 4.70 | 1.22 | 1.96 | / | / | / | / |
| S524 | 3.70 | 1.83 | 1.05 | / | / | / | / |
| S530 | 4.65 | 1.87 | 1.04 | / | / | / | / |
| S531 | 3.70 | 1.82 | 2.5 | / | / | / | / |

Table S3: Clinical data of two NB patients with relapsed plasma MYCN copy number

| Case | Age | Gender | Recurrence time | Stage | Primary lesion | Recurrent lesions | Recurrent manifestations | HVA/Crn | VMA/Crn | NSE (ng/mL) |
| --- | --- | --- | --- | --- | --- | --- | --- | --- | --- | --- |
| 1 | 1 | female | 1 | IV | Chest | primary lesion / lymph nodes | recurrent fever | 11.90 | 70.72 | 198.60 |
| 2 | 5 | male | 5 | IV | left adrenal gland | primary lesion / marrow | lower extremity pain | 10.85 | 96.02 | 49.00 |

Table S4: Detection of MNA status strategy based on NB tissue and plasma

| Sample | Detection method | Detection time | Detection operation | Sample acquisition | Sensitivity | Specificity |
| --- | --- | --- | --- | --- | --- | --- |
| NB tissue | FISH | >24h | Difficulty | Difficulty | Consistent | Consistent |
| Plasma | RT-PCR | 2h | Easy | Easy |  |  |
